# Msuite2: All-in-one DNA methylation data analysis toolkit with enhanced usability and performance

**DOI:** 10.1016/j.csbj.2022.03.005

**Published:** 2022-03-10

**Authors:** Lishi Li, Yunyun An, Li Ma, Mengqi Yang, Pengxiang Yuan, Xiaojian Liu, Xin Jin, Yu Zhao, Songfa Zhang, Xin Hong, Kun Sun

**Affiliations:** a School of Chemical Biology and Biotechnology, Peking University Shenzhen Graduate School, Shenzhen 518055, China; b Institute of Cancer Research, Shenzhen Bay Laboratory, Shenzhen 518132, China; c BGI-Shenzhen, Shenzhen 518083, Guangdong, China; d School of Medicine, South China University of Technology, Guangzhou 510006, Guangdong, China; e School of Medicine, Sun Yat-sen University, Guangzhou 510080, China; f Women's Hospital, School of Medicine, Zhejiang University, Hangzhou 310006, Zhejiang, China; g Guangdong Provincial Key Laboratory of Cell Microenvironment and Disease Research, Department of Biochemistry, School of Medicine, Southern University of Science and Technology, Shenzhen, 518055, China

**Keywords:** CpG dinucleotide, Bisulfite treatment, Data visualization

## Abstract

DNA methylation is an important epigenetic regulator that plays crucial roles in various biological processes. Recent developments in experimental approaches and dramatic expansion of sequencing capacities have imposed new challenges in the analysis of large-scale, cross-species DNA methylation data. Hence, user-friendly toolkits with high usability and performance are in urgent need. In this work, we present Msuite2, an easy-to-use, all-in-one, and universal toolkit for DNA methylation data analysis and visualization with high flexibility, usability, and performance. Msuite2 is among the fastest tools in read alignment (in particular, it runs as much as 5x faster than its predecessor, Msuite1) with low computing resource usage. In addition, Msuite2 shows both balanced and high performance in terms of mapping efficiency and accuracy, demonstrating high potential to facilitate the investigation and application of large-scale DNA methylation analysis in various biomedical studies. Msuite2 is freely available at https://github.com/hellosunking/Msuite2/.

## 1 Introduction

DNA methylation is a widely recognized epigenetic modification existing in most species, and it is known as a crucial molecular regulator responsible for many biological processes [1], [2], [3]. DNA methylation is believed to correlate with gene silencing, and its strong cell-type specific pattern suggests its importance in tissue development, cell identity determination [4] and promises broad applications in translational fields, such as cancer diagnosis and tumor subtyping [5], [6], [7], [8], [9]. In mammals, the most important and pervasive DNA methylation category is 5-methylcytosine (5mC) regulated by various methyltransferase proteins, which mostly occurs in CpG dinucleotides [10]. Currently, whole-genome bisulfite sequencing, or WGBS, is the most widely used and comprehensive experimental approach to investigate 5mC profile, and it offers base-pair resolution methylation status of all cytosines in the genome [11], [12]. In WGBS, the bisulfite treatment converts the unmethylated cytosines to thymines while leaves the methylated ones unchanged, and therefore allows the differentiation of methylated and unmethylated cytosines. However, the bisulfite treatment procedure alters the original DNA sequence, which reduces the complexity of the DNA and makes the data analysis task complex and time-consuming [13]. In addition, recent technical development of novel experimental assays, such as Tet-assisted pyridine borane sequencing (TAPS) [14], have utilized different strategies in differentiating methylated and unmethylated cytosines, thus imposing new requirements in data analysis. Therefore, easy-to-use, high-performance data analysis toolkits are urgently needed to accelerate DNA methylation investigations.

In past years, other researchers and we have developed a handful of bioinformatics tools for WGBS data analysis [13], [15], [16]. For instance, we have integrated quality control, read alignment, methylation call, and data visualization features in one package, Msuite (referred as Msuite1 hereafter) [15]. As bisulfite treatment procedure interrupts the reverse-complimentary relationship of the forward and reverse chains of the genome, most of the current tools utilize a 2-step alignment strategy (i.e., align the reads to the Watson and Crick chains separately), which requires lots of computational efforts and is error prone in handling reads with multi-hits [17]. To keep up with the emerging technical upgrades and tremendous improvement of current sequencers, in this paper we present Msuite2, the successor of Msuite1, for integrated DNA methylation data analysis with improved usability, as well as balanced and enhanced performance. Msuite2 package with testing dataset is freely available at https://github.com/hellosunking/Msuite2/.

## 2 Materials and methods

**Fig. 1:**
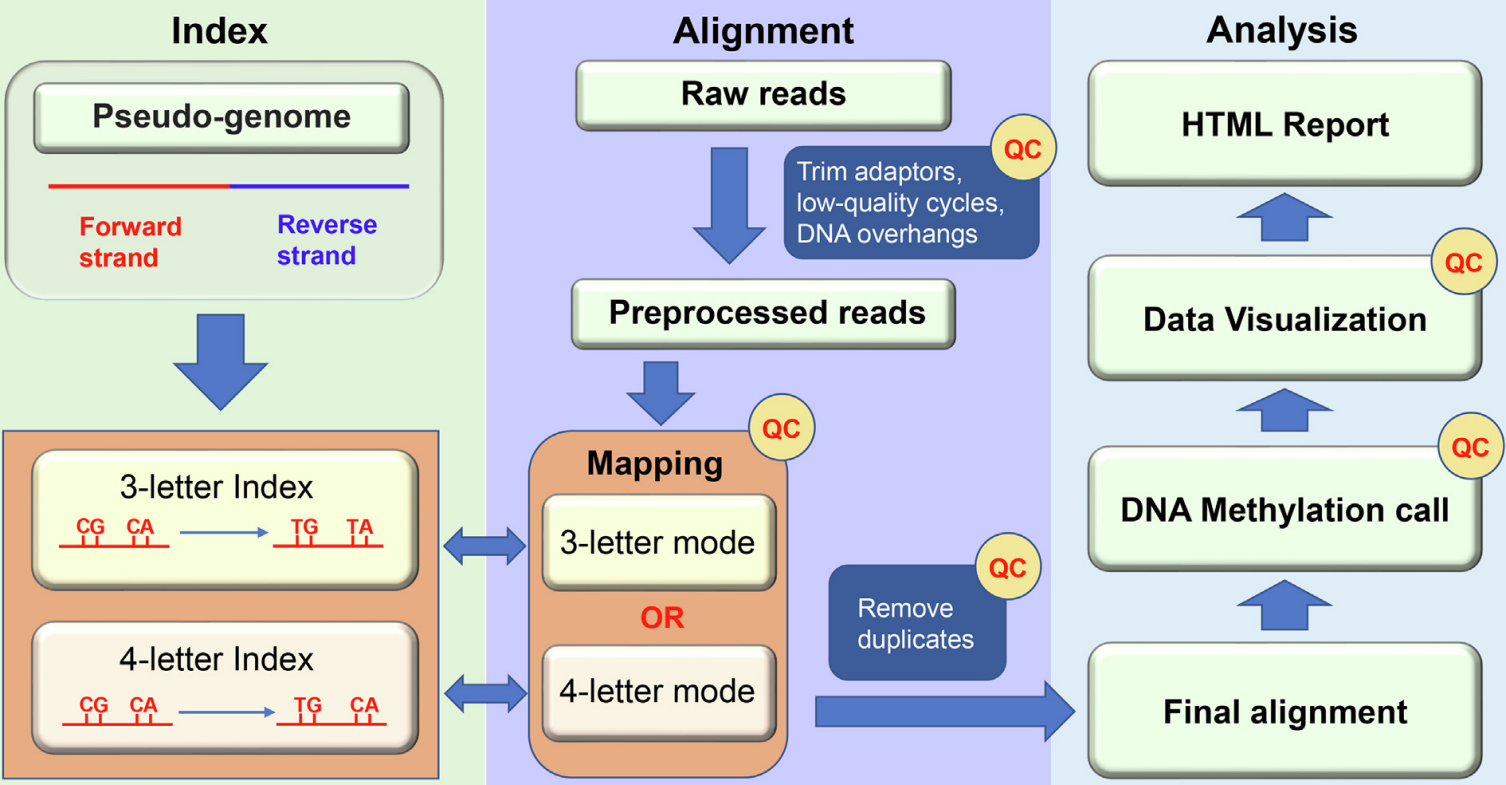
**Schematic workflow of Msuite2.** Msuite2 has packaged sequencing read preprocessing, alignment, DNA methylation call and data visualization.

Fig. 1 illustrated the schematic workflow of Msuite2 and Table 1 showed the major features of Msuite2 in comparison with representative tools in this field (i.e., Msuite1 [15], Bismark [18] and BWA-meth [19]). Msuite2 is composed of 4 major components: quality control, read alignment, methylation call, and data visualization. Like its predecessor, Msuite2 employs Ktrim [20] to get rid of sequencing adapters and low-quality cycles, but it offers additional flexibility that it allows the users to further discard any length of heading and/or tailing cycles to minimize the impact of single-strand overhang problem in DNA termini [21], which issue introduces bias during DNA end repair step thus affecting correct DNA methylation inference, and better supports library preparation protocols that add additional sequences to the insert DNA [22]. In addition, Msuite2 keeps the two analysis modes as in Msuite1: the 3-letter mode (i.e., convert all cytosines to thymines) is a universal analysis mode similar to the current tools, while the 4-letter mode (i.e., only convert cytosines in CpG context to thymines) provides optimized support for emerging bisulfite-free DNA methylation assays such as TET-assisted pyridine borane sequencing (TAPS) [14], [15]. As demonstrated in Msuite1, the exclusive 4-letter analysis mode in Msuite2 and Msuite1 could largely improve the performance (including speed and accuracy) of analyzing sequencing data generated using TAPS-like protocols [15]. For read alignment, Msuite2 not only supports Bowtie2 as used in Msuite1 [23], but also adds support of Hisat2 (which runs much faster than Bowtie2) [24] as the underline aligner. Moreover, the most remarkable improvement in read alignment strategy of Msuite2 is that it combines the forward and reverse strands to form a pseudo-genome, which allows it to map the reads only once against this pseudo-genome for speed-up and more proper handling of reads with multi-hits. After alignment, Msuite2 identifies and removes the PCR duplicates, and then call the DNA methylation levels for all CpG sites in the genome. Notably, detailed quality control metrics are recorded during each analysis step. Finally, Msuite2 collects the key statistics and generates various visualizations related to data quality and analysis results into a self-explanatory HTML format report to the users.

**Table 1:** Comparison of major features between Msuite2 and current tools.

	Msuite2	Msuite1	Bismark	BWA-meth
Underlying aligner	Bowtie2/Hisat2	Bowtie2	Bowtie2/Hisat2	BWA
Align mode	3-/4-letter	3-/4-letter	3-letter only	3-letter only
Read preprocessing#	Yes	Yes	No	No
Flexible read cycles	Yes	No	Partially^$^	No
Quality control	Yes	Yes	No	No
Methylation call	Yes	Yes	Manually	No
Data visualization	Yes	Yes	No	No
Indel support	Yes	Yes	Yes	Yes
Multiple-file support	Yes	Yes	Yes	Yes
Sequencing mode	PE/SE	PE/SE	PE/SE	PE/SE
Output format	BAM	SAM/BAM	BAM	SAM
Parallelization	Yes	Yes	Yes	Yes

# Read preprocessing includes trimming of sequencing adaptors and low-quality cycles; ^$^Bismark allows the users to skip the heading cycles.

## 3 Results

### 3.1 Benchmark performance evaluation

**Fig. 2:**
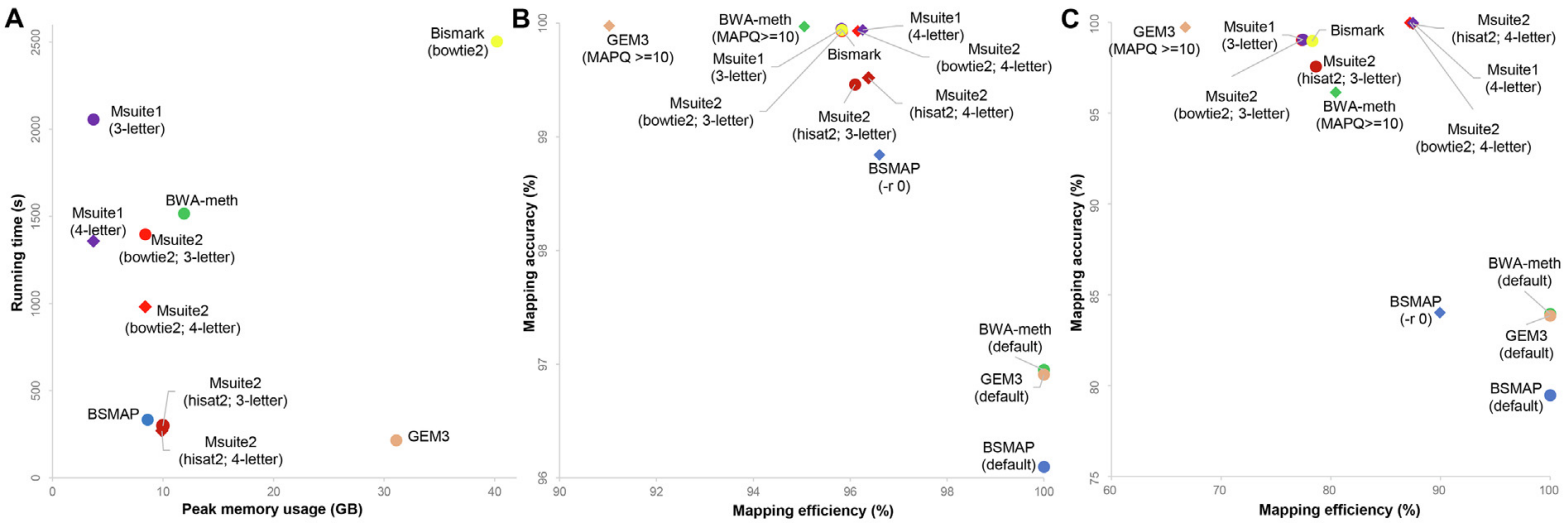
**Benchmark evaluation results of Msuite2 and current tools.** (A) running time and peak memory usage (8 threads), (B) mapping accuracy and efficiency on 10 M *in silico* paired-end 100 bp reads, (C) accuracy and efficiency on 10 M *in silico* paired-end reads simulated in CT-rich regions. For BWA-meth, BSMAP, and GEM3, default and alterative parameters were both tested. The reads were simulated following TAPS protocol to enable the 4-letter mode of Msuite2 and Msuite1; results in 10 repeat experiments were averaged and shown. MAPQ stands for mapping quality score.

We performed a benchmark evaluation of Msuite2, Msuite1, and the latest versions of current mainstream tools (i.e., Bismark [18], BWA-meth [19], BSMAP [25], and GEM3 [26]) using *in silico* generated reads. The results were summarized in Fig. 2 and Supplementary Figure S1-S2. Hence, Msuite2 supports the usage of Hisat2 as the underline aligner and achieves a similar level of running speed as BSMAP and GEM3, and is as much as 5x faster than Msuite1, BWA-meth, and Bismark, making it one of the fastest tools in read alignment. In the meantime, Msuite2 requires a moderate level of computational memory, which is much lower than GEM3 and Bismark (Fig. 2A), suggesting that Msuite2 can even run on a common personal computer.

Fig. 2B showed the mapping efficiency (i.e., the proportion of raw reads that are mapped) and accuracy (i.e., the proportion of alignment results that are correctly mapped) of these tools. Msuite2, Msuite1 and Bismark show comparable mapping accuracies, which are higher than BWA-meth, BSMAP and GEM3 when running with default parameters. We tried to adjust the parameters of BWA-meth, BSMAP and GEM3 to improve the mapping accuracy and found that when we obtained comparable mapping accuracies to Msuite2, the mapping efficiencies of these tools decreased at the same time (Fig. 2B), which result indicates that these tools may suffer from unbalanced efficiency and accuracy (i.e., default parameters show high efficiency but suboptimal accuracy, and fine-tuned parameters rescue the accuracy while sacrifice the efficiency). Notably, the 4-letter mode of Msuite2 and Msuite1 shows higher accuracy and efficiency than the 3-letter mode. We further benchmarked these tools using reads simulated from the CT-rich regions. Such regions are enriched in regulatory elements so they are important in DNA methylation studies [15]. The results were summarized in Fig. 2C, which showed a very similar trend to Fig. 2B and demonstrated more prominent advantage of Msuite2.

In addition, Msuite2 package provides more features besides read alignment. In fact, Msuite2 has integrated the whole data analysis pipeline, including quality control, methylation call and data visualization, into 1 command. For instance, before alignment, Msuite2 automatically trims the sequencing adaptors and low-quality cycles (i.e., data preprocessing) to improve the alignment efficiency and accuracy; after read alignment, Msuite2 performs methylation call, analysis statistics summary, and data visualization. Hence, Msuite2 shows balanced and enhanced performance in data analysis, as well as multifunctionality and improved usability.

### 3.2 Output example of Msuite2

To demonstrate the usage of Msuite2, we collected a real WGBS dataset from ENCODE (human transverse colon tissue with accession number ENCSR156JXJ) [27], which contains around 262 million paired-end 150 bp reads. We then analyzed the data using Msuite2 on an ordinal computing server equipped with two 16-thread Xeon CPUs and standard 64-bit Linux operating system. Utilizing Hisat2 as the underline aligner, Msuite2 completed the read preprocessing and alignment within 1 h, and the whole analysis was finished within 70 min.

**Fig. 3:**
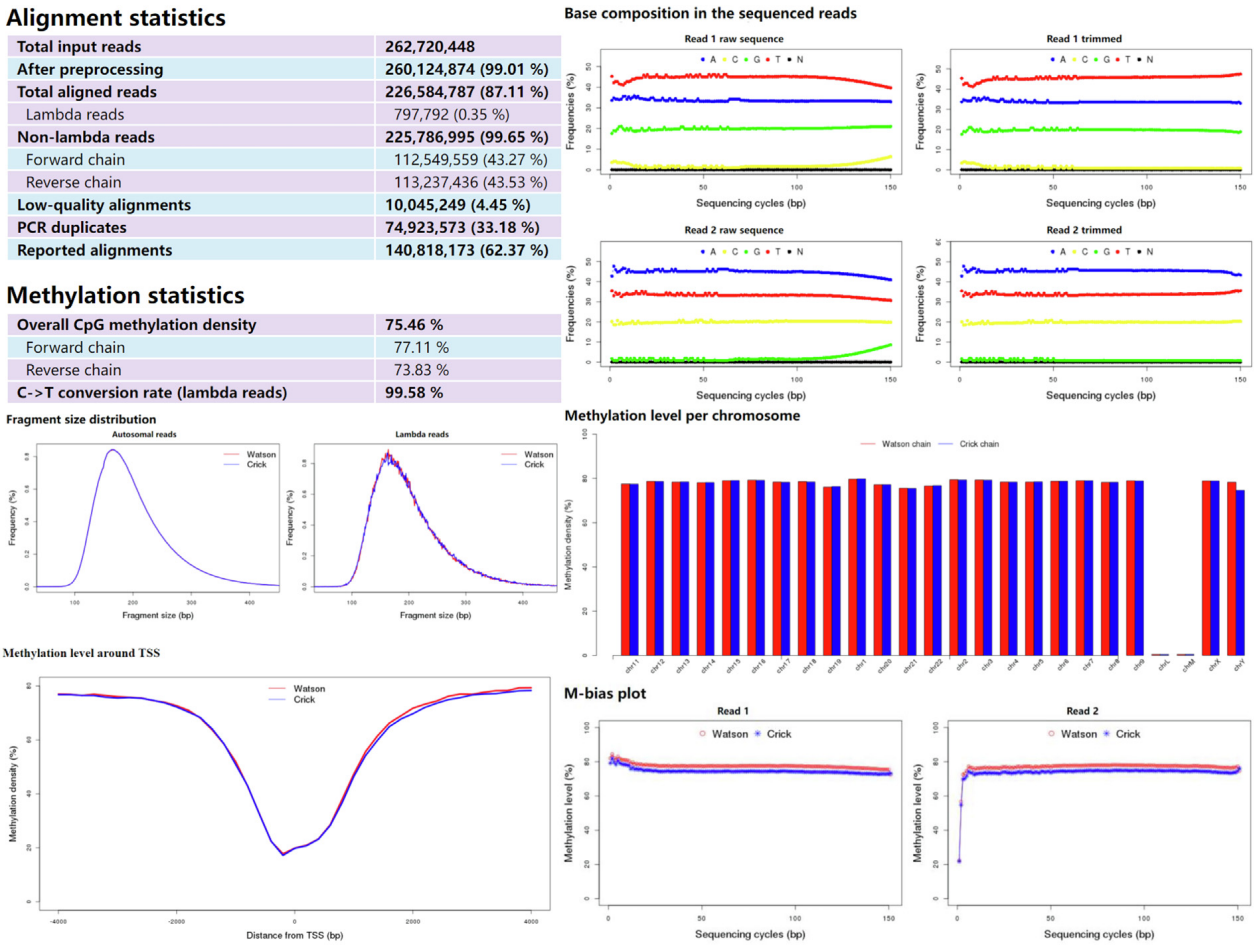
**Output example of Msuite2 on real data.** Msuite2 reports the key statistics of the analysis, as well as various figures to help the users inspect the quality of the data.

The output of Msuite2 includes aligned reads in standard BAM format, DNA methylation calls for each CpG site, as well as an easy-to-follow HTML report shown in Fig. 3. Compared to Msuite1, the HTML report has been redesigned to provide improved flexibility and optimized presentation of the data analysis for the users. Msuite2 reports the key statistics of the analysis, including mappable reads, duplicates, overall DNA methylation level as well as conversion rates (automatically estimated using lambda spike-ins). Particularly, Msuite2 reports the number of low-quality alignments, PCR duplicates and final reported alignments separately; such detailed statistics could help the users to inspect the quality of their DNA library and optimize experiment designs. Furthermore, Msuite2 presents fruitful visualizations to the users to help them inspect the quality of their data. For example, firstly, the base compositions before and after trimming offer the users to check the quality of sequencing data, adapter contaminations, and the effectiveness of Msuite2′s preprocessing step; secondly, unlike Msuite1, Msuite2 separately plots the DNA-of-interest and lambda spike-ins in fragment size distribution, as in some scenarios they could be rather different (e.g., plasma cell-free DNA [28]); finally, the methylation level of each chromosome and around transcript start sites (which are known to be a valley-like signal) could serve as a quick quality-control of the DNA methylation data. Moreover, Msuite2 provides M−bias across each sequencing cycle; one could observe aberrantly low DNA methylation level at the heading cycles of read 2, which could be (partially) caused by the DNA overhang issue.

### 3.3 Application on real biological data

**Fig. 4:**
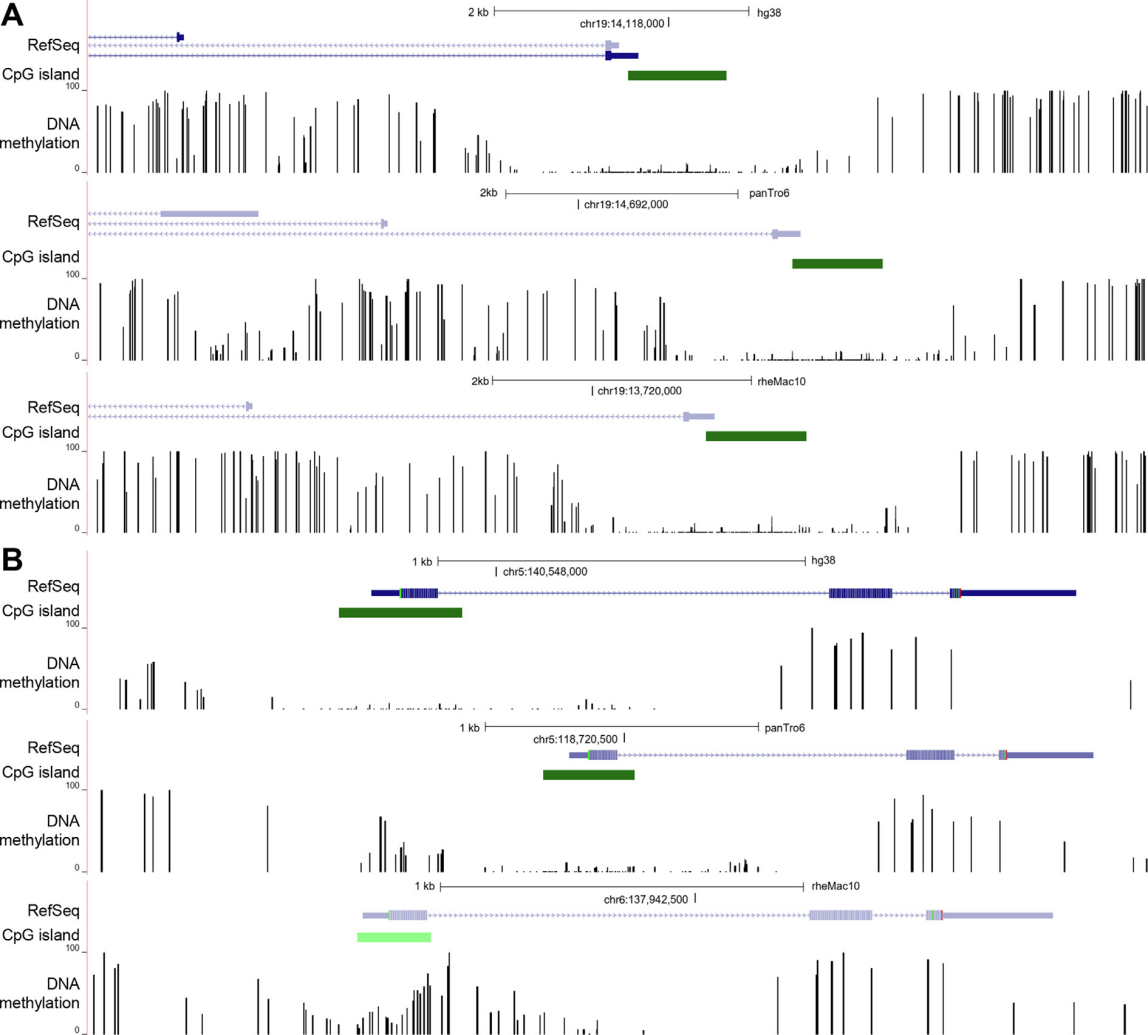
**DNA methylation profiles of the liver tissue in human, chimpanzee, and macaque.** (A) *PRKACA* gene, (B) *EIF4EBP3* gene.

To further illustrate the application of Msuite2 in real biological studies, we analyzed a large-scale, multi-species WGBS dataset generated by Blake *et al.,* [29]. We used Bowtie2 as the underline aligner, and Msuite2 finished the whole analysis (including quality control, read alignment, methylation level inference, and data visualization) of 15.3G reads (4.6G, 5.2G, and 5.4G reads for human, chimpanzee, and macaque, respectively) within 54 h. In particular, sequencing reads preprocessing and alignment were completed in 18.3, 17.6, and 17.7 h, respectively for human, chimpanzee, and macaque data while it may take > 96 h for the existing tools (estimated based on Fig. 2A). Fig. 4 showed the methylation profile of *PRKACA* (protein kinase cAMP-activated catalytic subunit alpha) promoter, which gene is highly conserved among the 3 species [29]; in contrast, *EIF4EBP3* (eukaryotic translation initiation factor 4E binding protein 3) gene promoter is hypo-methylated in human and chimpanzee liver but hypermethylated in macaque liver, suggesting that this gene may play roles during speciation.

## 4 Discussion

In this paper, we present Msuite2, an all-in-one, easy-to-use package for DNA methylation data analysis. Through integration of quality control, read alignment, methylation call and data visualization, Msuite2 aims to provide a 1-command package to lower down the entry of DNA methylation data analysis. Msuite2 only requires the users to input their raw sequencing data, and then performs the whole analysis with a user-friendly analysis report. Besides the high usability, Msuite2 also shows balanced and enhanced performance over the current tools. In addition, Msuite2 supports both the conventional 3-letter analysis mode (i.e., convert all cytosines to thymines) and a unique 4-letter analysis mode (i.e., only convert cytosines in CpG dinucleotides to thymines) which is specifically designed for emerging bisulfite-free assays, and we demonstrated that the 4-letter mode does possess advantages in analyzing such data, such as higher speed and accuracy. In conclusion, Msuite2 could serve as a valuable toolkit to facilitate the large-scale DNA methylation analysis in various molecular biological studies, especially for bench scientists.

## CRediT authorship contribution statement

**Lishi Li:** Methodology, Formal analysis, Writing – original draft. **Yunyun An:** Methodology, Formal analysis. **Li Ma:** Formal analysis. **Mengqi Yang:** Formal analysis, Writing – review & editing. **Pengxiang Yuan:** Formal analysis. **Xiaojian Liu:** Formal analysis. **Xin Jin:** Validation, Formal analysis, Funding acquisition. **Yu Zhao:** Validation, Formal analysis. **Songfa Zhang:** Conceptualization, Validation, Formal analysis, Supervision. **Xin Hong:** Conceptualization, Validation, Formal analysis, Supervision, Funding acquisition. **Kun Sun:** Conceptualization, Methodology, Software, Formal analysis, Writing – original draft, Writing – review & editing, Supervision, Funding acquisition.

## Declaration of Competing Interest

The authors declare that they have no known competing financial interests or personal relationships that could have appeared to influence the work reported in this paper.
