# Supplementary material for: Msuite2: All-in-one DNA methylation data analysis toolkit with enhanced usability and performance
Source: Comput Struct Biotechnol J. 2022 Mar 10;20:1271–6. doi: 10.1016/j.csbj.2022.03.005 (PMC8918723; doi:10.1016/j.csbj.2022.03.005)
Supplement: Supplementary data 1 [file mmc1.docx]

**Msuite2: all-in-one DNA methylation data analysis toolkit with enhanced usability and performance**

Lishi Li, Yunyun An, Li Ma, Mengqi Yang, Pengxiang Yuan, Xiaojian Liu, Xin Jin, Yu Zhao, Songfa Zhang, Xin Hong, Kun Sun

This file contains supplementary Figure S1 and S2.


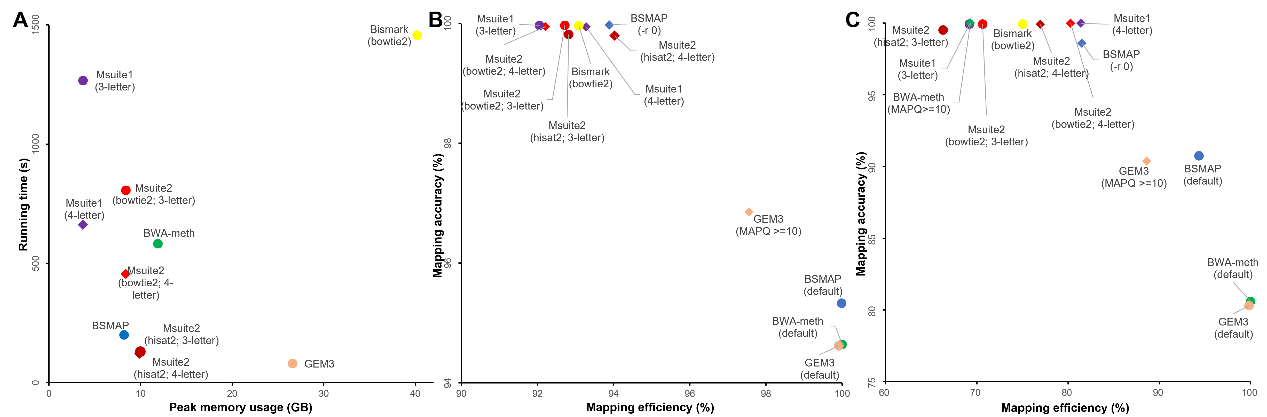


**Figure S1. Benchmark evaluation results of Msuite2 and current tools using single-end data.** (A) running time and peak memory usage (8 threads), (B) mapping accuracy and efficiency on 10M *in silico* single-end 100 bp reads; (C) accuracy and efficiency on 10M *in silico* single-end reads simulated in CT-rich regions. For BWA-meth, BSMAP, and GEM3, default and alterative parameters were both tested. The reads were simulated following TAPS protocol to enable the 4-letter mode of Msuite2 and Msuite1; results in 10 repeat experiments were averaged and shown. MAPQ stands for mapping quality score.


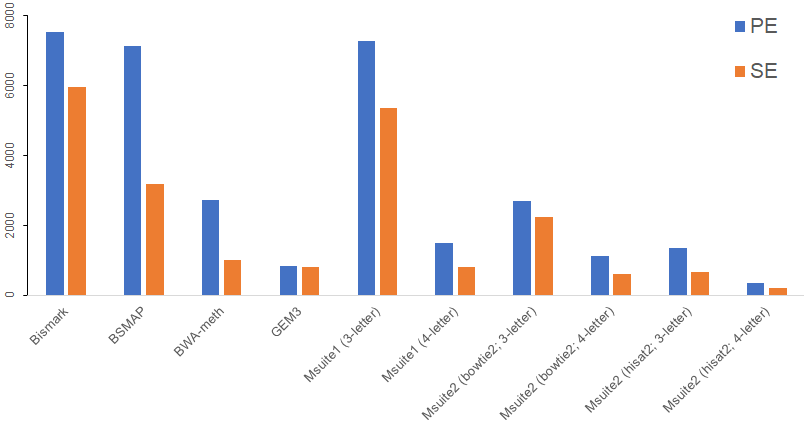


**Figure S2. Running time of Msuite2 and current tools on CT-rich reads.** Blue and yellow bars represent results on 10M paired-end 10 bp and 10M single-end 100 bp data, respectively.
